# How to deal with COVID-19 epidemic-related lockdown physical inactivity and sedentary increase in youth? Adaptation of Anses’ benchmarks

**DOI:** 10.1186/s13690-020-00432-z

**Published:** 2020-06-03

**Authors:** Irène Margaritis, Sabine Houdart, Youssef El Ouadrhiri, Xavier Bigard, Anne Vuillemin, Pascale Duché

**Affiliations:** 1 French Agency for Food, Environmental and Occupational Health & Safety (Anses), Nutrition Risk Assessment Unit, 14 rue Pierre et Marie Curie, 94701 Maisons-Alfort, Cedex France; 2 International Cycling Union (UCI), Allée Ferdi Kübler 12, 1860 Aigle, Switzerland; 3 Université Côte d’Azur, Laboratoire Motricité Humaine Expertise Sport Santé (LAMHESS), 261 Boulevard du Mercantour, BP 3259, 06205 Nice, Cedex 3 France; 4 Université de Toulon, Unité de recherche Impact de l’activité physique sur la santé (IAPS), CS 60584, 83041 Toulon, Cedex 9 France

**Keywords:** COVID-19, Sedentary lifestyle, Behaviors, Screens, Children, Adolescents, Physical activity

## Abstract

Faced with the spread of the SARS-CoV-2 virus, regulatory measures aiming to prevent interpersonal contaminations have been undertaken and among these, lockdown. Due to strong restrictions out-of-home movements, we hypothesize that overall physical activity will decrease and sedentary behavior increase. This could result in highest exposure to the well-known risk related to insufficient physical activity. To mitigate physical inactivity and sedentary behaviors health-related risks related to children and adolescents lockdown and school closure, Anses (French Agency for Food, Environmental and Occupational Health & Safety) has adapted, within the first days of the public authorities’ prescription, its former benchmarks. This paper supports and comments Anses’ Opinion by raising the questions of whether, why, and how to deal with short- or medium-term lockdown-related physical inactivity and sedentary behavior increases. Short-term and unknown long term-impacts on mental health and well-being, physical fitness and eating behaviors clearly appearing for children and adolescents as being the main issues of concern are highlighted. Targeting the compensations of the physical inactivity increase, the types, frequencies and durations of physical activity, are adapted to restricted environment. Sedentary behavior limitation and frequent interruptions becomes a priority. Overall, considering children and adolescents, the emerging risk justifies proposing specific adaptations and type of activities in order to ensure maintaining health underpinned, at least partly, by physiological equilibrium and physical fitness and avoid the installation of new unhealthy habits or routines that young people could keep after lockdown.

## Background

If children and adolescents seem relatively protected from COVID-19 infection, their health is affected by prolonged lockdown that may, in itself, trigger risks to the health and well-being of young people.

The major role of physical inactivity (PI) and sedentary behaviors (SB) in health-related risks and most chronic disease prevention has justified the publication of science-based benchmarks. These benchmarks for reducing the daily time spent in a seated position and for practicing regular physical activities (cardiorespiratory capacities, muscle building, balance and flexibility) have been updated by Anses (French Agency for Food, Environmental and Occupational Health & Safety) in 2016 [1], considering usual social life and daily activities.

Since 17 March 2020, faced with the spread of the SARS-CoV-2 virus, responsible for the COVID-19 disease, regulatory measures aiming to prevent interpersonal contaminations, have been undertaken within the framework of the “state of health emergency”. France, like most countries, imposes strict lockdown measures for an announced period of several weeks that will probably be followed by school closure and spontaneous self-restrictions until population feel new confidence for outdoor activities. In this context, Anses has carried out the expert appraisal: “Assessment of the risks of reduced physical activity and increased sedentary levels in lockdown situations” linked to the COVID-19 epidemic [2]. This opinion relates to the healthy young population on lockdown in application of the French regulation – this issue is of international concern. Countermeasures are to date solely based on expert elicitation. Based on Anses’ opinion, updated by the most recent knowledge – especially on the current outbreak – this article raises the questions of whether, why, and how to deal with short- or medium-term lockdown-related PI and SB increase in young children and adolescents.

## Main text

### Whether

Lockdown, as a maintenance of people in a space of restricted and closed volume, considerably limits movement, affects the spontaneous physical activity (PA) usually related to the necessities of daily life outside the home. The effect of these negative impacts associated with an insufficient level of PA of moderate to high intensity and a high level of PI or SB is widely recognized. These effects have been identified and characterized for several populations, as a part of the report and opinion on the PA and SB benchmarks published in 2016 [1]. As an evidence, the strong restrictions out-of-home movements increase the exposure to these dangers, inducing an increase in associated health risks. The lockdown and the school closure prescribed by the public authorities during the current pandemic, and for several weeks, lead children and adolescents to restrict their movement. To compensate for the decreased spontaneous PA related to the necessities of daily out-of-home life and avoid or at least alleviate the effects of restricted daily movement, the types of PA, their frequencies and durations have to be adapted.

### Why?

#### Because physical health and fitness

Home care for children and adolescents is known to decrease the level of PA and increase in sedentary time, in particular screen time [2].

In the context of the SARS-CoV-2 pandemic, lockdown is not total (certain outings remain authorized) even if remaining associated with measures of “social distancing”. However, to characterize the dangers and hypothesize the risks, we have considered the worst-case scenario (this is to say that people do not exert outdoor activities) and postulate that the lockdown period will remain limited to a few weeks – despite the uncertainties as to the exact duration of this period and the conditions of the further social life recovery. To date, due to the lack of data, the so-called risk cannot be assessed, and the work has been realized based on strong hypothesis of modified exposures.

With the hypothesis that lockdown results in a decrease in PA in young people, the situation can be similar to a situation of detraining, even in young children (7 years old), strength and balance were assessed as weak after 8 weeks of detraining, i.e. stopping PA at school with or without strength training twice a week [3].

#### Because chronic disease risk

If PI and SB are associated with the risks of overweight, obesity and cardiometabolic complications, in return, regular PA reduces these risks and is associated with early mortality prevention helping preventing the onset of most chronic diseases [1]. Several experimental and observational studies emphasize that SB, and in particular “TV time”, are associated with higher obesity and cardio-metabolic risks in children and adolescents [4]. In addition, other studies also highlight the associations between the use of screens (especially television and smartphones) and eating habits that can have a negative effect on health [5].

A sedentary lifestyle is a risk factor for chronic diseases and increased mortality. The context of lockdown promotes these SB and can lead to an increase in time spent sitting or lying down (apart from sleeping), the effects of which can be deleterious on health, including mental health.

It is known that the duration-factor of SB, the intermission plays a major preventing role. During a 3-h sitting position, regular break every 30-min (with 3 min of moderate intensity walking) in children of 7–11 years has been shown to lower insulin response and plasma free fatty acids to the consecutive meal [6] compared to uninterrupted SB. The very regular break in sedentary times by promoting PA, even if it involves low intensity and short-term PA is the first step in modifying SB profile [1] and is a point to emphasize. Overall, both considering the daily time spent in a sitting position and respecting breaks for prolonged periods will potentially have effects on eating behaviors at risk of metabolic complications in children and adolescents.

#### Because diet

SB are associated with eating behavior leading to higher energy intake. To illustrate, a study [7] analyzed the level of PA and the duration of SB on the specific effects of food intake. Energy intake is higher (+ 350 kcal) when SB times increase (corresponding to − 100 kcal of energy expenditure).

In addition, lockdown-related increases in SB can worsen food intake having potentially adverse health effects (e.g. increased frequency of snacking, consumption of energy-dense foods) [8] especially the consumption of sugar-sweetened beverages whose metabolic risks are proven [9, 10]. Moreover, an unbalanced diet could even worsen especially vitamin D status which is impacted by the lack of outdoor activities. In this regard, Anses points out the importance of ensuring sufficient intake by a balanced diet including vitamin D-rich foods.^1^

Overall, to reduce both nutritional and metabolic risks, the interruption of SB is of prime importance. Having healthy nutritional habits is essential in this context, necessarily associated with measures to reduce PI [9–11].

#### Because mental heath

The recent lockdown in China during the current COVID-19 epidemic showed high levels of anxiety and stress. Anxiety is associated with an increase in cortisol secretion whom rhythm of secretion is disturbed, and the quality of sleep is affected. The recent experience in China shows that children in the younger age group (3–6 years) were likely to manifest symptoms, such as clinginess and fear that family members could contract the infection. Children aged 6 to 18 years were more likely to show inattention [12] or have affected mental health as by other traumatic experiences [13]. Another study in young people aged 14–35 years reported 40.4% of psychological problems and 14.4% of Post-Traumatic Stress Disorder symptoms [14]. Expecting upcoming data, these emerging signals encourage considering the role of lifestyle including getting enough PA – to limit PI – and SB limitation which can both contribute to mental health protection [1], especially in children and adolescents in the context of school closure and lockdown.

### How?

To move toward new benchmark proposals, the benchmarks developed in 2016 for children and adolescents [1] – as they are underpinned by the assessment of health risks related to PA and SB – have been adjusted to benchmarks suitable for lockdown (as summarized in Table 1). To reach this goal, the adjustments have taken into account the reduction of daily activities imposed by high restriction for people to leave home and engage in regular activities (e.g., school, work) or access to places of PA practice (e.g. parks, walking trails, etc.), even near the home.

**Table 1:** Anses’ benchmarks adapted in the context of lockdown

Duration and frequency	Intensity level	Exercises to adjust depending on the type of housing (with or without outdoors)
**PA for children under 6 years**		
At least 3 h of PA throughout the day:	Keep PA both frequent and playful	Activities (play, run, jump) in safe spaces indoor and outdoor (if available)
That being 15 min/h for 12 h of awake time		Make board games more active by introducing balance and stretching challenges
		Use a mat for somersaults
**PA for children and adolescents from 6 to 17 years (including muscle strenght training activities)**		
At least 60 min of PA throughout the day	At least two 10 min sessions of moderate intensity	Use household items, as well as stairs and furniture to train the muscles
Record duration of PA		Make board games more active by introducing balance and stretching challenges
Divide the daily duration of physical activity in sessions of 10 to 20 min		
Muscle strength training activities twice a week	Moderate intensity	Exercises using the body weight or a wall (stand and sit, push, lean on), small equipment (elastic bands, weighted arm bands, dumbbells) or muscle training gear (rowing machine, training bicycle)
		Use household items as exercise equipment (bottles, broom...)
**Limit SB of children under 6 years and children and adolescents from 6 to 17 years**		
Limit sedentary time.	Reduce SB more frequently by breaking up prolonged sitting time	Limit the increasing leisure-based and passive screen time
Ideally stand up every 30 min		
Limit leisure-based and passive screen time		

Anses considers here the relevant handles from it opinion [1] to put them into perspective of lockdown and proposes the appropriate benchmark statements.

The adaptation of PA practice rules is imperative for physiological (maintenance of muscular functions, bone health, body composition, etc.) and psychological health (recreational activities allowing tightening the family ties in a general context of anxiety).

#### From former to adapted benchmarks: physical activity

The PA benchmarks for children under 6 years are *at least 3 h per day of PA,* i.e. *15 min/h for 12 h of wakefulness* remain applicable during lockdown. However, the PA benchmarks for children aged 6 to 17 are *at least 60 min/d of moderate to high intensity PA* need to be adjusted.

In children aged 6 to 17 years, the PA benchmarks should maintain a total duration of 60 min accumulated per day (PA duration has to be measured). This duration can be split into a period of 10 to 20 min several times a day (to be distributed). A moderate intensity has to be maintained at least over two 10-min periods and muscle reinforcement has to be practiced twice a week.

Therefore, in children and adolescents, dynamic activities will be favored, as well as balance and flexibility exercises.

PA has to be gradually settled into everyday life with progressive intensity and frequency, as well as adapted to the current level of fitness and health status. It is more important to comply with continuity and regularity principles as a priority rather than focus on the intensity of the PA.

Anyway, even if the occurrence of COVID-19 symptoms are less frequent and milder in young people, it cannot be excluded that a febrile state requires stopping moderate to very high PA. The risk of myocarditis is inherent in any viral infection; these inflammations of the myocardium can lead to arrhythmia, which can turn out to sudden death from exercise. For safety reasons, PA has to be stopped if fever or other suspected clinical signs of COVID-19 (fatigue, dry cough, etc.) occur. The risk of spreading the virus in the cardiac tissue such as adenovirus, parvovirus, and herpesvirus is well known, but whether this risk of complication also exists for coronaviruses, including SARS-CoV-2 is unknown. Absolute rest for fever is an intangible rule.

Moreover, even if under lockdown conditions, the intensities and durations of cardiorespiratory exercises are not likely to reach levels such that one can fear a fall in immune defenses in the hours that follow the activity, it is reasonable to ensure that exercises are not exhausting.

#### From former to adapted benchmarks: sedentary behaviors

SB in this context is a major concern, especially for young people. Sedentary lifestyle benchmarks for children until 17 years: *Limit the total daily duration of sedentary activities while awake; limit the duration of each sedentary activity, so as not to exceed 1 h continuously for children under 6 and 2 h for children aged 6–17 years.*

In children under 6 years and in children 6 to 17 years, the sedentary lifestyle benchmarks should be adapted during the lockdown period to ensure limit sedentary time while maintaining a playful and regular PA As well as all populations, it has to be emphasized that the pace of breaking inactivity by limiting time sitting continuously has to rise every 30 min. Because of the well-known, and to date documented effects, parents have to ensure prevention in the increase in recreational and passive screen time that can be consecutive to lockdown being aware to strongly limit time screen to less than 60 min in young children (2–4 years) as recommended by WHO [15].

The full effectiveness of these benchmarks, as prioritized in Table 2, also depends on compliance with general rules and habits of healthy living in confined spaces, including mental health preservation, sleep quality, and diet.

**Table 2:** Priorities for PA and SB benchmark in a lockdown situation (adapted from [2])

	Children < 6 y	Children and adolescents 6-17 y
SB limitation	+++	+++
Muscle strength	+	++
Cardiorespiratory	+	++
Balance	++	+
Flexibility	+	+

## Conclusions

In the context of the actual lockdown, and considering the necessity of empowering people to preserve their own health, Anses has proposed adapted benchmarks in the continuation of the former [1] to allow, or at least facilitate, the adaptation to the specific context of lockdown for all the populations.

Not much is known about the long-term mental health effects of lockdown on children and adolescents, but it can be hypothesized that some of them could adopt or reinforce unhealthy behaviors. Considering children and adolescents, the emerging risk justifies proposing specific adaptations and type of activities in order to avoid the installation of unhealthy habits and ensure maintaining health underpinned, at least partly, by physiological health and physical fitness. Moreover, attention payed to PI and SB limitation is a unique leverage to deal with already observed outcomes in a Chinese study: it allows to relieve children’s distress and address their concerns regarding the negative condition they are experiencing [12].

The situation of lockdown inevitably induces adoption of a sedentary lifestyle and eating habits that are not favorable to health. SB such as watching TV and use of computer/games are relatively stable behaviors which track moderately in childhood and adolescence [16] and from early childhood to later in life. In the same way, PI appears as a stable behavior throughout childhood and adolescence. Reducing continuous sedentary times by frequent interruptions may help to alleviate the hypothesized harmful effects of even reduced PA. The probable increase in the time dedicated to leisure screens used passively can be considered as being a priority vigilance point to ensure the limitation of these durations. Indeed, PI in itself is an unhealthy habit with consequences on adulthood [17].

Children and adolescents are likely to adopt adverse behavioral and eating habits on health whose reversibility is to date unknown but can at least be prevented.

Overall, it is proposed that promotional and videos could motivate children to have a healthy lifestyle at home by increasing PA, having a balanced diet, regular sleep pattern [18]. However, to be safe, this elicitation has to be considered on the basis of evidence-based benchmark involving the scientific and educational community.

In usual conditions, the link between socioeconomic status and SB which are negatively associated [19] is well known. This relationship is similar with PA [1]. During the current COVID-19 epidemic the high levels of anxiety and stress or depression, the social capital of people on lockdown were inversely correlated too [20]. As the role of social inequalities with regard to the practice of PA and SB is already well known and the probability of perpetuating them or even worsening them in a lockdown situation is obviously high.

To our knowledge, studies on the effect of pandemic lockdown in children and adolescents are scarce. However, despite the existence of additional factors (linked to duration, social ties, etc.), it has been considered that the behavior of children staying at home during school holidays or at weekends can be partially transposed to this assessment of the lookdown-related risk [2]. As schools can actively promote a health-conscious schedule, including PA, school closure and home confinement leads de facto to the privation of this promoting effect as an additional factor to the space restriction for movement. In this context, families need help through benchmarks to ensure the continuance of both PA motivation and SB limitation.

It has to be kept in mind, that, as an evidence, COVID-19 appears to be preferentially virulent among adults with existing comorbidities including obesity, hypertension, and diabetes which prevalence is known, at least partly, to be linked with lifestyle. We hypothesize this health crisis has the potential to trigger further outcomes. The very special situation we are facing could affect durably some behaviors, especially in young people. Consequently, we have to be aware as to keep sufficient pressure on the preventive effects of PA and limited SB. Since the beginning of the epidemic and the establishment of lockdown in a large number of countries, a part of the scientific community reiterates that modifiable lifestyle factors like diet and PA should not be marginalized. The main concern could be the emergence of new unhealthy habits or routines that young people could keep after lockdown and what these adjusted benchmarks could help to avoid. Hitherto, the lack of experience of this situation does not allow not neglect the risk.

Ensuring adequate PA for the duration of the lockdown would thus help to limit the consequences of PI and SB, and, in the present context, to maintain habits after the lockdown has ended. It will also help the resumption of usual activities or the acquisition of new ones and ensure a better resilience of the way out of lockdown.

## Data Availability

Not applicable.

## Abbreviations

Anses: Agence nationale de sécurité sanitaire de l’alimentation, de l’environnement et du travail (French Agency for Food, Environmental and Occupational Health & Safety)
PA: Physical activity
PI: Physical inactivity
SB: Sedentary behaviors

## Footnotes

1 https://www.anses.fr/en/content/ensuring-sufficient-vitamin-d-intake-through-diet-during-lockdown
